# In silico structural homology modelling of EST073 motif coding protein of tea *Camellia sinensis* (L)

**DOI:** 10.1186/s43141-020-00038-6

**Published:** 2020-07-19

**Authors:** K. H. T. Karunarathna, N. H. K. S. Senathilake, K. M. Mewan, O. V. D. S. J. Weerasena, S. A. C. N. Perera

**Affiliations:** 1 Institute of Biochemistry, Molecular Biology and Biotechnology, University of Colombo, Colombo, Sri Lanka; 2 Current address: Department of biosystems Technology, Faculty of Technology, University of Ruhuna, Matara, Sri Lanka; 3 Department of Biotechnology, Faculty of Agriculture and Plantation Management, Wayamba University of Sri Lanka, Makandura, Gonawila, Sri Lanka; 4 Department of Agricultural Biology, Faculty of Agriculture, University of Peradeniya, Peradeniya, 20400 Sri Lanka

**Keywords:** Blister blight disease, EST-SSR, Homology modeling, Molecular markers, Tea

## Abstract

**Background:**

Tea (*Camellia sinensis* (L). O. Kuntze) is known as the oldest, mild stimulating caffeine containing non-alcoholic beverage. One of the major threats in south Asian tea industry is the blister blight leaf disease (BB), caused by the fungus *Exobasidium vexans* Masse. SSR DNA marker EST SSR 073 is used as a molecular marker to tag blister blight disease resistance trait of tea. The amino acid sequences were derived from cDNA sequences related to EST SSR 073 of BB susceptible (TRI 2023) and BB resistant (TRI 2043) cultivars. An attempt has been made to understand the structural characteristics and variations of EST SSR 073 locus that may reveal the factors influencing the BB resistance of tea with multiple bioinformatics tools such as ORF finder, ExPasy ProtParam tools, modeler V 9.17, Rampage server, UCSF-Chimera, and HADDOCK docking server.

**Results:**

The primary, secondary, and tertiary structures of EST SSR 073 coding protein were analyzed using the amino acid sequences of both BB resistant TRI 2043 and BB susceptible TRI 2023 tea cultivars. The coding amino acid sequences of both the cultivars were homologous to photosystem I subunit protein (PsaD I) of *Pisum sativum*. The predicted 3D structures of proteins were validated and considered as an acceptable overall stereochemical quality. The BB resistant protein showed CT repeat extension and did not involve in topology of the PsaD I subunit. The C terminal truncation of BB resistance caused the formation of hydrogen bonds interacting with PsaD I and other subunits of photosystem I in the modeled three-dimensional protein structure.

**Conclusions:**

*Camellia sinensis* EST 073 SSR motif coding protein was identified as the PsaD I subunit of photosystem I. The exact mechanism of PsaD I conferring the resistance for blister blight in tea needs to be further investigated.

## Background

Tea, *Camellia sinensis* (L*.*) O. Kuntze, is the second most popular, healthy non-alcoholic beverage in the world. It is an economically important tree crop, grown in several countries in Asia and Africa. Globally, Sri Lanka is the third-largest producer and the 2^nd^ largest exporter of tea [1] with its popular brand “Ceylon Tea”, playing a key role in the international tea trade.

Blister blight leaf disease (BB), caused by the obligatory fungal pathogen, *Exobasidium vexans* Masse (Basidiomycetes) is one of the most devastating biotic constraints, commonly found in a majority of tea plantations in south Asia including Sri Lanka, India, Indonesia, Bangladesh, Thailand, Nepal, Vietnam, Cambodia, and Japan [2]. The BB leaf disease causes approximately 25 to 30% crop loss annually depending on the agro-ecological region (AER) of Sri Lanka [3]. The disease infection also causes a reduction of the quality of black tea by changing the composition of leaf biochemical components such as polyphenols, catechins, and enzymes which highly influence the quality of black tea [4].

Presently, the control of this disease is solely based on chemical means, where spraying of Cu-based fungicides directly on to the foliage, before infection, being the recommended practice. The disease is very common in major tea growing areas of Sri Lanka throughout the year, and the repeated application of Cu-based fungicides may lead to chemical residues in the end product “Black tea.” Though tea, is a popular healthy beverage, exceeding maximum residual levels (MRLs) of pesticides, heavy metals, and other chemical impurities, leads to a non-tariff trade barrier in exporting and consumption of tea [5]. Therefore, to overcome the said constraints and also to maintain the quality of the symbol “Ceylon Tea”, the development of resistant cultivars to BB disease would be the most effective and sustainable approach to control the disease.

Tea is a perennial crop, which requires 20–25 years to develop a new improved cultivar and therefore, the application of marker-assisted selection (MAS) techniques would be highly desirable to increase the efficiency and effectiveness of the breeding program. Bulk segregant analysis (BSA) approach has successfully been applied to identify a SSR DNA marker EST SSR 073 to tag blister blight disease resistance trait using a segregating population derived from the two parents: TRI 2043 (resistant cultivar) × TRI 2023 (susceptible cultivar) [6]. EST SSR 073 motif correlates with the photosystem I subunit D (PsaD I) and identifying the structural model of a protein of the motif is one of the key points for understanding the underlying biological mechanism at a molecular level. The available knowledge on the structure and the role of PsaD I protein is scarce. The experimental elucidation of the tertiary structure of a protein is a huge and a difficult endeavor [7]. The X-ray crystallography or nuclear magnetic resonance techniques (NMR), which are applied to identify the tertiary structure of a protein, are time consuming and expensive [8, 9]. However, the “*In silico* homology modeling” provides an alternative application to predict the 3D structure of proteins with better validation. Homology modeling is known to be one of the best and extensively used computational methods to generate three-dimensional structures when there is more than 35% sequence identity between the known protein structure (template) and the unknown protein structure [10–13].

In silico homology modeling has been successfully applied to predict the structure of Matrix metalloproteinase 25 (MMP 25) and it can be used as a target for the inhibition of airway remodeling in asthma disease by using in silico drug designing methods [14]. Furthermore, an acceptable protein structure of nif A which is involved in nitrogen fixation of rhizobial strains, has been identified and validated by using in silico structure homology modeling [15]. In silico characterization of ChiLCV coat proteins of *Begomovirus* in chilli aided in the development of strategies to control *Begomovirus* disease of crops [16]. Vascular wilt disease of tomato caused by *Fusarium oxysporum f*. sp. *lycopersici* is controlled by targeting a novel candidate protein FOXG_04696 which has been developed by homology modeling [17].

With the above background, molecular modeling of EST SSR 073 motif coding protein was the objective of the current study to provide a topology for revealing protein folding and functional structure which would help in understanding the blister blight fungal infection for combating the disease.

## Methods

### DNA sequence of EST SSR 073 motif

The EST SSR 073 motif containing cDNA sequence of blister blight disease resistant tea cultivar TRI 2043 (BBR) (GenBank accession no: MT303817) [18] and the DNA sequence of EST SSR 073 motif of blister blight disease susceptible tea cultivar TRI 2023 (BBS) (GenBank accession no: MT303818) [6] were retrieved. The sequences of BBR and BBS were aligned with BLASTn program [19].

### Amino acid sequence analysis and template retrieval

All possible open reading frames (ORFs) for both the nucleotide sequences were identified by ORF finder (NCBI) [20]. Amino acid sequences derived by conceptual translation of each of the ORFs were used as the query for searching homologous sequences using BLASTp [21] against uniprotKB/swissprot database to identify potential orthologs [22]. The search was repeated against Protein Data Bank (PDB) and the amino acid sequence which contained the putative conserved domain and showed the highest sequence similarity and the lowest *E* value, was selected for structure modeling.

### Homology modeling and energy minimization

PsaD subunits have been reported to possess N (1Met to GLY 90) and C (Asp171 to Gly 193) terminal unstructured domains which are involved in the assembly of photosystem I super complex [23, 24]. Accordingly, the three-dimensional (3D) structure of the identified proteins were built using modeler V 9.17 [25] using the crystal structure of PsaD subunit of *Pisum sativum* photosystem I super-complex (PDB ID: 5l8r_D) [26] as the template and viewed by UCSF Chimera [27]. The generated model of *C. sinensis* PsaD-like protein was superimposed on the PasD subunit of 5l8r in energy minimized state while keeping the rest of the complex fixed. Superimposition was carried out using Matchmaker function of UCSF-Chimera [28, 29]. Energy minimization was carried out using AMBER force field [30–32] in chimera with 100 steps of steepest descents followed by 10 steps of conjugate gradients.

### Protein model validation

The quality of generated models was validated with respect to backbone and side chain geometry. To validate protein backbone quality, Ramachandran plot [33] was generated using Rampage server (http://mordred.bioc.cam.ac.uk/~rapper/rampage.php) and the backbone quality was validated by analyzing φ and ψ angles using Ramachandran plot. Further; VERIFY3D, ERRAT, PROVE, PROCHECK, AND WHATCHECK [34] servers were used to analyze the overall quality of the model.

### Structural comparison of modeled proteins

Optimized energy minimized protein models generated for the sequence derived from BB susceptible TRI 2023 and the sequence derived from BB resistant TRI 2043 were superimposed using Matchmaker function of UCSF Chimera and RMSD (root mean square deviation) value was obtained. Further, structural comparison was carried out by superimposing and RMSD evaluation against the template protein that was used to generate the protein models.

### Validation of physiological parameters

Structure-function relationship of the derived protein models was further validated using ProtParam tool of ExPASy Proteomics Server [35] for various parameters such as estimated half-life, theoretical pI, instability index, aliphatic index, and grand average of hydropathicity (GRAVY). The values were compared with the protein sequence used as the template as well as the BB susceptible and resistant genotypes.

### Molecular docking

Crystal structure of PSI-I complex of *Pisum sativum* (PDB ID: 5l8r) was retrieved from RCSB-PDB. Subunits that interact with PsaD were retained and other subunits were removed using UCSF-Chimera. Modeled PsaD was docked against the binding site of the complex by HADDOCK docking server [36]. Docking results were viewed using UCSF Chimera. Default parameters were used for docking process and energy (E) values of each docking event were obtained. For comparative analysis, docked complexes were compared with the interactions of the PSI complex of *Pisum sativum.*

## Results

### Nucleotide sequence analysis and comparison

The best aligning nucleotide sequence of the Genbank database for both sequences was *Camellia sinensis* photosystem I reaction center subunit II, chloroplastic-like (LOC114287061), and mRNA (sequence ID: XM_028230326.1) derived from shuchazao tea cultivar of China. The EST SSR 073 motif containing cDNA sequence of blister blight disease resistant tea cultivar TRI 2043 displayed 99.07% identity with 0.0% *E* value by covering 92% query coverage. The cDNA sequence of blister blight disease susceptible tea cultivar TRI 2023 showed 99.33% identity with 0.0% *E* value by covering 92% query coverage. Further, nucleotide sequences obtained for both BBS and BBR had over 80% identity with the coding sequence of *Diospyros kaki* photosystem I subunit D-I (PsaD-1) mRNA (ID: KX871204.1) with *E* value of 5e− 117.

**Fig. 1:**
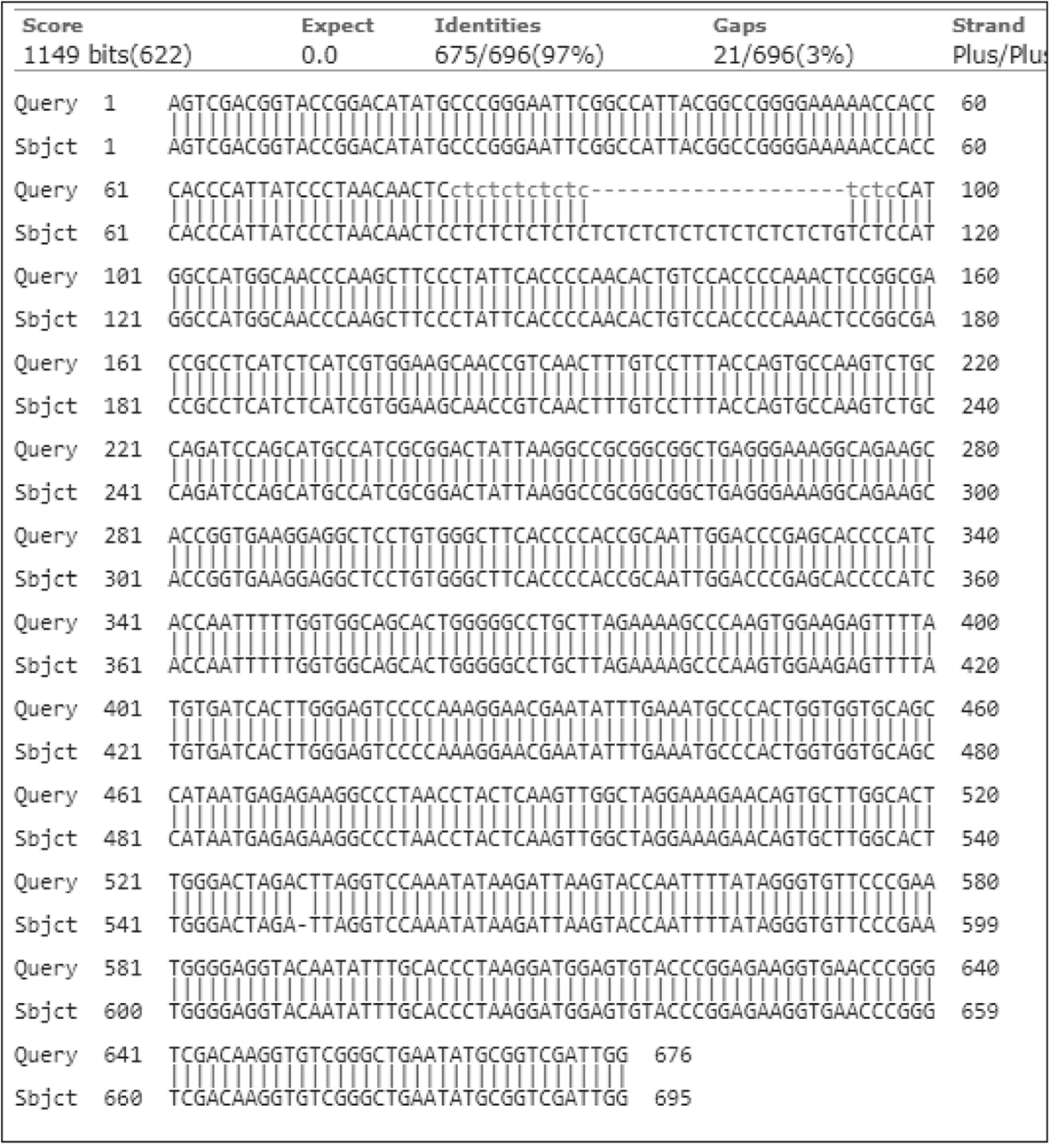
Global pairwise alignment of EST 073 loci related to BB resistant and BB susceptible nucleotide sequences. Query denotes the BBS sequence and sbjct denotes BBR sequences.

Pairwise alignment of BBS and BBR sequences indicated 20 microsatellite CT repeat extension in 5′UTR of BBR and a single nucleotide deletion at 552 bp (deletion of C) (Fig. 1).

### Amino acid sequence analysis

**Fig. 2:**
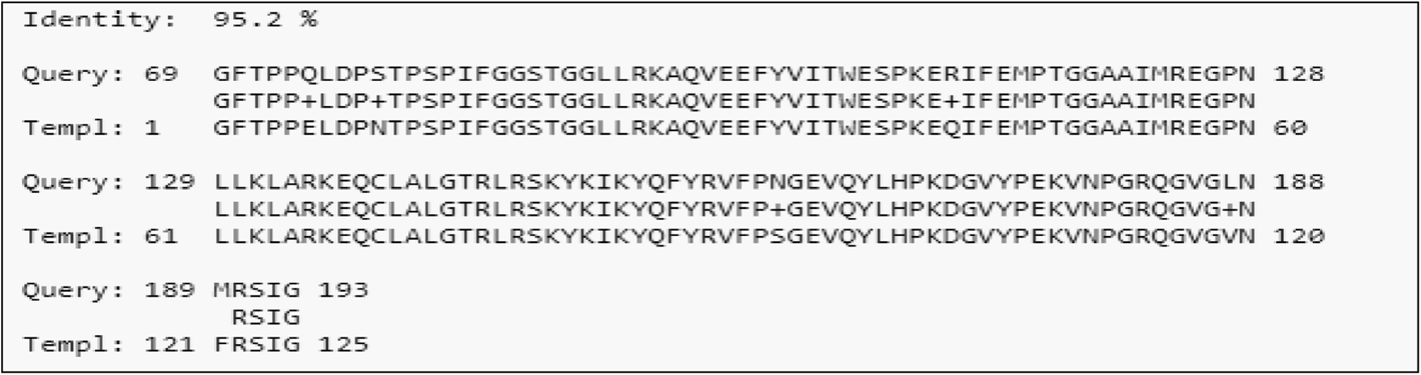
Protein sequence alignment of BBS and *P. sativum*

The amino acid sequence obtained from the longest ORF of BBS sequence was 193 amino acid in length and had 97% similarity and 95.2% identity over the full length of PsaD subunit of *Pisum sativum* photosynthesis complex I (Fig. 2).

**Fig. 3:**
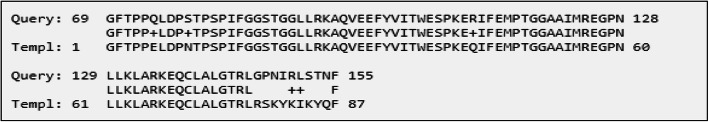
Protein sequence alignment of BBR and *P. sativum*

The BBR protein sequence had a 86.2% identity and 89.1% similarity with the PsaD subunit of *P. sativum*. Both the nucleotide and protein sequence comparison clearly showed a frame shift mutation (single nucleotide deletion) leading to truncated C terminal caused by the single nucleotide deletion of BBR at 552 bp as shown in the sequence alignments in Fig. 3. The search was repeated against PDB database and the crystal structure of *P. sativum* photosynthesis complex I was retrieved for homology modeling.

### Homology modeling and structure comparison

**Fig. 4:**
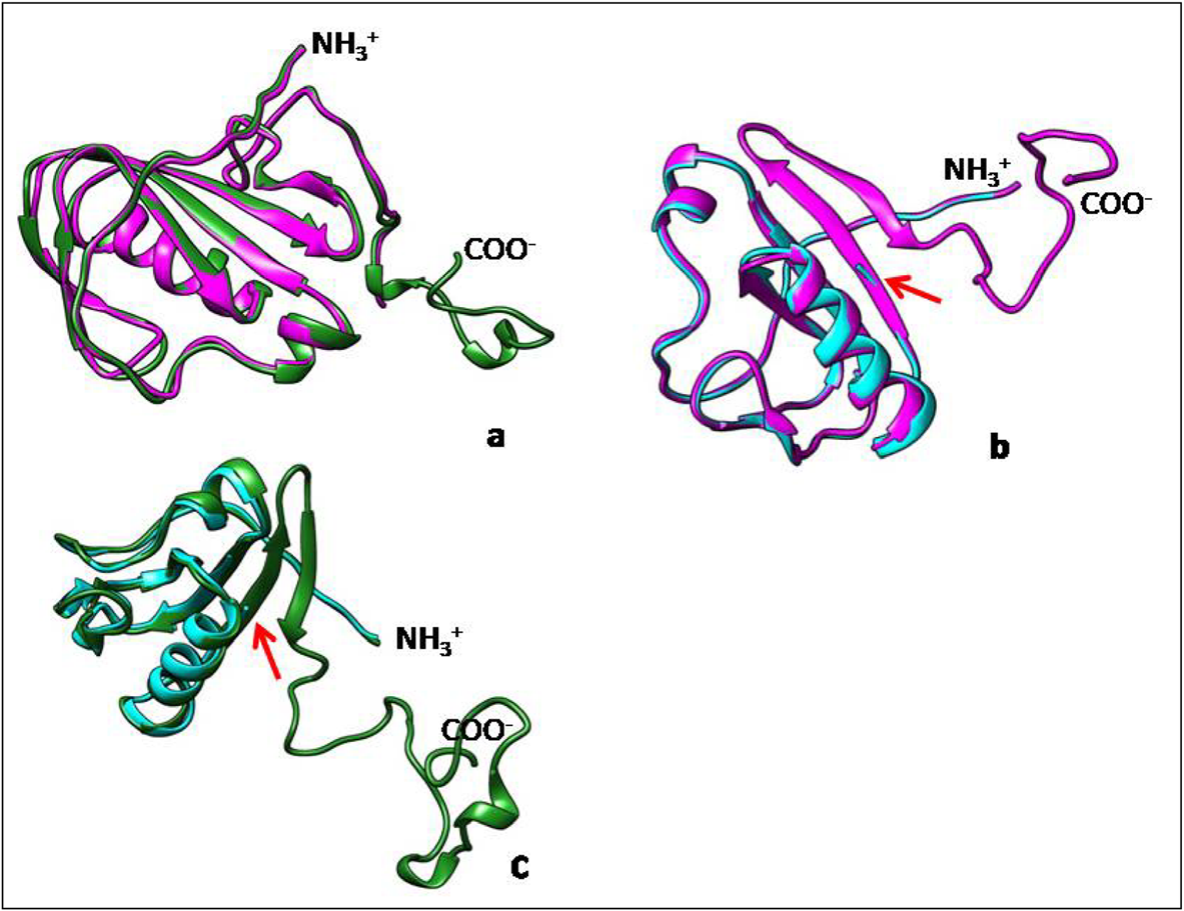
Superposition of the generated models with the PsaD subunit of *P. sativam* (red colored arrows indicate the truncation of PsaD subunit of BBR). **a-** PsaD subunits from BBS (magenta) and *P*. *sativum* (green). **b-** PsaD subunits from BBR (cyan) and BBS (magenta). **c-** PsaD subunits from BBR (cyan) *P*. *sativum* (green)

After homology modeling and energy minimization superimposed model of the BBS sequence retained the same fold and domain structures as the PsaD subunit of *P. sativum* with a RMSD value of 0.530 Å. More importantly, all amino acids that form H bonds with other subunits of the complex were conserved in *P. sativum* and *C. sinensis* (Fig. 4a). The model generated from BBR sequence clearly showed truncation of the C terminus, completely eliminating the anti-parallel beta strands and the C terminal unstructured domain (Fig. 4b, c). Rest of the structure was the same as the BBS and had an RMSD value of 0.616 Å when compared to aligning region of PsaD of *P. sativum*.

### Structure validation

**Fig. 5:**
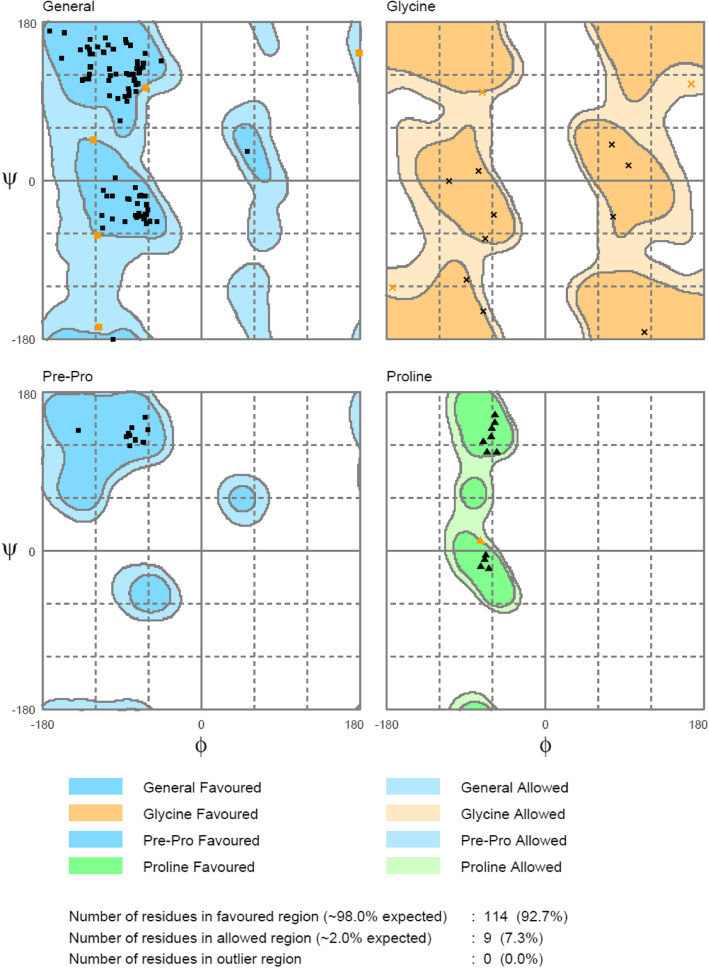
Ramachandran plot statistics of BBS model

**Fig. 6:**
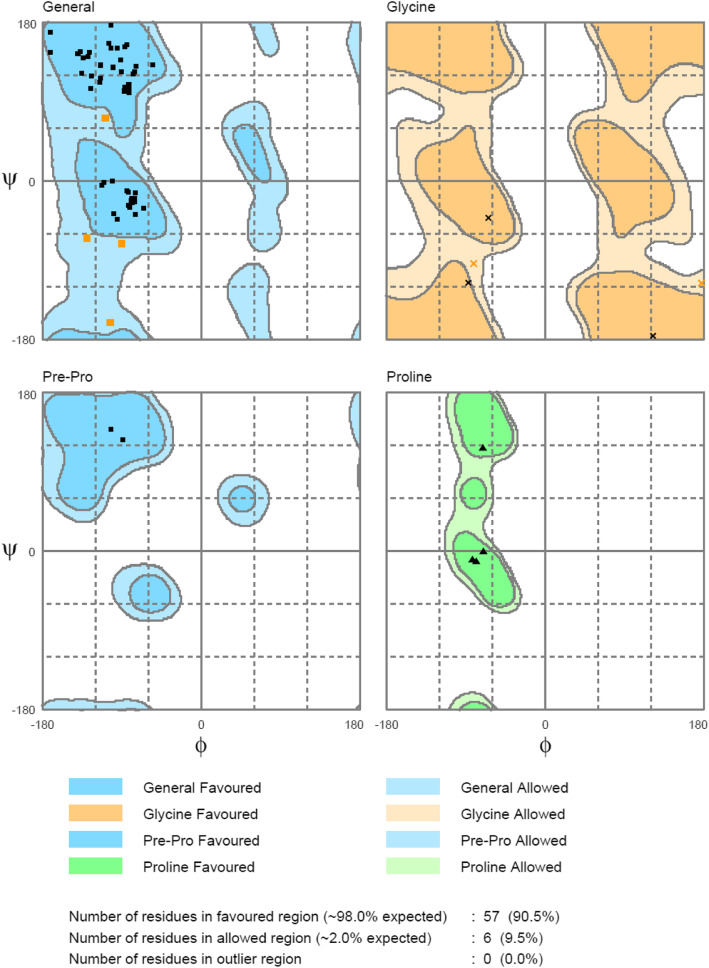
Ramachandran plot statistics of BBR model

After energy minimization, the models generated for BBS and BBR were showed good overall stereochemical quality as expected for modeled with high sequence identity with the template [37–39]. The BBS model had no residues in the outlier region, while 92.7% of residues lied in the favored region (Fig. 5), and BBR had 90% of its residues in the favored region, while no residues were found in the outlier region (Fig. 6).

**Fig. 7:**
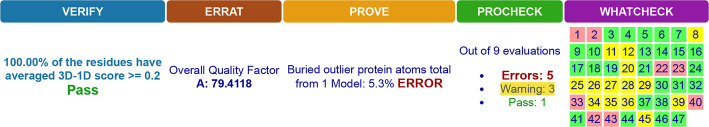
Summary of BBS model quality analysis by VERIFY, ERRAT, PROVE, PROCHECK, and WHATCHECK servers

**Fig. 8:**
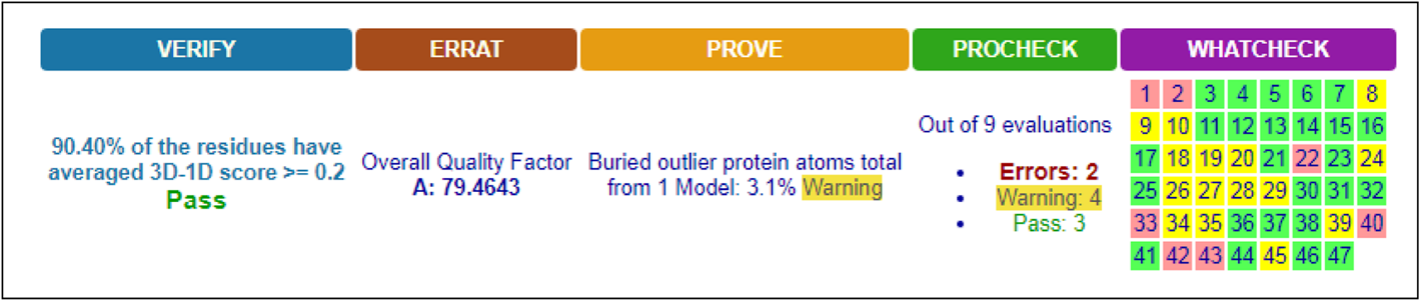
Summary of BBR model quality analysis by VERIFY, ERRAT, PROVE, PROCHECK, and WHATCHECK servers

Further quality analysis using VERIFY3D, ERRAT, PROVE, PROCHECK, AND WHATCHECK servers indicated a good overall quality of both BBS (Fig. 7) and BBR (Fig. 8) homology models.

Generated structures and the PsaD subunit of *P*. *sativum* were further compared with physicochemical parameters such as theoretical isoelectric point (pi), estimated half-life, instability index, aliphatic index, and the grand average hydropathicity (GRAVY) of BBR, BBS, and PsaD subunit of *P*. *sativum* as given in Table 1.

**Table 1:** Physiological parameters of PsaD subunits

Parameter	*P. sativum*	BBR	BBS
Estimated half-life	30 H	30 H	30 H
Theoretical pI	9.46	10.10	9.91
Instability index	47.74	60.65	45.55
Aliphatic index	72.87	69.04	69.17
GRAVY	− 0.513	− 0.310	− 0.419

### Molecular docking and interaction analysis

**Fig. 9:**
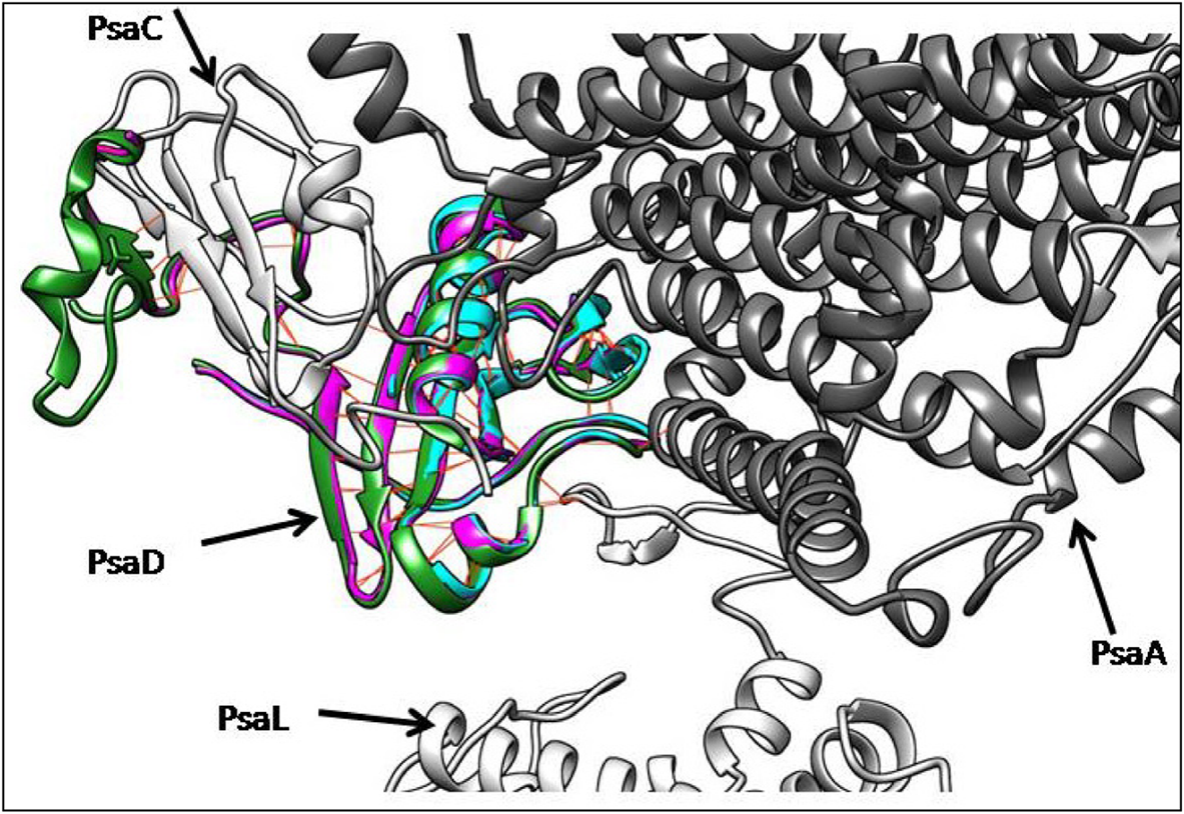
Superposition of docking poses of PsaD subunits form BBS, (magenta), BBR (cyan), and *P. sativum* (green). Brown color lines show H bonds.

For comparative analysis, docked complexes were compared with the interactions of the PSI complex of *Pisum sativum* (Fig. 9)*.* Interaction analysis showed that in both *P. sativam* and BBS PsaD s, all residues that involve in H bonding with PsaA, Psa C, and Psa L to be conserved and showing similar interaction patterns. However, BBR proteins possessed a C terminal truncation which prevents PsaD from interacting with PsaC.

## Discussion

The EST SSR 073 motif containing cDNA sequences of both TRI 2043 and TRI 2023 display significant similarity with PsaD I subunit nucleotide sequence of Chinese tea cultivar shuchazao by confirming the reliability of DNA sequence data were used in the study. Furthermore, the DNA sequences displayed high similarity with the *Diospyros kaki* photosystem I subunit D-I. Accordingly, both of the sequences were identified as putative PsaD subunit of photosystem I of *Camellia sinensis*. In almost all plants, the Psad I gene exists as a single-copy gene [40].

The CT extension of BBR does not change the sequence and the structure of protein. However, it may be involved in the post-transcriptional regulation of PsaD I expression. In fact, 5′UTR of most of the mRNA sequences contains regulatory structures such as hairpins [41]. Furthermore, the BBR sequence possesses a single nucleotide deletion at 552 bp (deletion of C) which leads to a truncation of ORF and the formation of a shorter protein product.

Homology modeling of the resulted amino acid sequences from BBS sequences using PsaD I of *P. sativum* as a template produced a three-dimensional structure of the protein which is very similar to the template used. PsaD I subunit is reported to be expressed as unfolded protein having a leader sequence and later undergoing proper folding when complexed with the rest of the subunit of photosystem I super complex. Also, PsaD subunits possess N terminal and C terminal domains having no rigid structure. However, these unstructured domains of PsaD I, particularly the C terminal unstructured domain has reported to be involved in forming H bonds with PsaC subunit of the complex. Therefore, energy minimization of the models generated for PsaD subunits of BBS and BBR sequences were carried out within the binding surface of PsaD I complex with the PsaD subunit.

After energy minimization, the 3D model obtained for BBS was almost identical to the secondary and tertiary structure of the PsaD I of *P*. *sativum*. Both of the generated models for BBS protein sequence and the PsaD I of *P. sativum* showed identical hydrogen bonding pattern with the other subunits of photosystem I super complex. When both models of BBS were docked against PsaD binding site of the photosynthesis complex, interestingly both protein models retained the same H bonding pattern within the complex by further confirming the adoption of the Psad I topology by them. Photosystem subunit D of photosystem I complex is hydrophobic and is exposed on stromal face of the thylakoid. The subunit interacts with ferredoxin in both cyanobacteria and eukaryotes [42].

The model generated for BBR had a C terminal truncation that eliminates the entire unstructured C terminal domain along with the C terminal anti-parallel beta sheets. It is unlikely that the BBR sequence would produce the functional protein because the interactions with PSaC is critical to maintain the stability of photosystem complex I. However, the N terminal extension of PsaD I in higher plants stabilizes the interactions with PsaC and rest of the photosystem I complex. Cross-linking study of barely, suggested that PsaD is stabilized with interaction between photosystem I H subunit [43] and Psa D is not tightly bound with photosystem I core [44]. Therefore, the C terminal truncation of the BBR may not involve in changing the stability of photosystem I complex and its main functions. Further, stability study of PsaD in *Synechocystis* has shown the reduced flavodoxin in, photosystem complex I without the PsaD subunit [45]. The mutation of BBR may be involved in so far unreported function and the predicted model may lead to discover the functions of mutated PsaD subunit.

The half-life of protein is the time it takes for half of the amount of protein in a cell to disappear after its synthesis in the cell. In this study, the half-life of all the proteins was 30 h. The instability index provides an estimate of the stability of the protein in a test tube. A protein whose instability index is smaller than 40 is predicted as stable, while a value above 40 predicts that the protein will be unstable [46]. The results from this study recorded instability index higher than 40 in all the three proteins (*P*. *sativum*, BBR, and BBS) indicating unstable properties. The aliphatic index of a protein is defined as the relative volume occupied by aliphatic side chains (alanine, valine, isoleucine, and leucine). If the aliphatic index is higher, the thermostability increases; therefore, the predicted proteins are thermostable. Isoelectric point is the condition of a solution where the amino acid produces the same amount of positive and negative charges and the ultimate charge will be zero. Isoelectric point (pI) of the three proteins was 9.4 to 10.1 and it seemed to be basic protein. The value of GRAVY spread between − 0.310 and − 0.514 and lower values are suggested to have good interactions between water and protein [47, 48].

In silico computational approach has been applied to predict protein structures of leaf rust disease resistance, and Lr 10 coding protein was identified as more resistant against the leaf rust disease of wheat [49]. However, the Psa D subunit has not been reported as associated with disease resistance up to now. Epicatechin (EC) and epigallocatachingallate (EGCG) are involved in BB disease resistance in tea [50]. Flavonoids biosynthesis pathway which synthesizes EC and EGCG is light sensitive and therefore, the allele may indirectly involve in the BB disease resistance.

## Conclusions

The EST SSR 073 motif flanking sequences of *Camellia sinensis* is conserved in the PsaD I subunit of photosystem I complex, and the developed in silico structures of homology proteins are reliable with their physicochemical parameters. When compared with BBS, CT repeat extension of BBR did not change the topology of PsaD I subunit but the single nucleotide deletion leads to C terminal truncation of BBR coding PsaD I subunit by preventing hydrogen bond interaction with other complexes of photosystem I. It can be recommended that more sequence data of EST SSR 073 motif flanking sequences in different tea cultivars and analyzing the protein model would lead to unravel the mechanism of BB resistance.

## Data Availability

Authors declare that all generated and analyzed data are included in the article.

## Abbreviations

BBS: Blister blight susceptible DNA sequence of TRI 2023
BBR: Blister blight resistance DNA sequence of TRI 2043
BB: Blister blight
SSR: Simple sequence repeat
DNA: Deoxyribonucleic acid
EST: Expressed sequence tag
PSaDI: Photosystem I subunit D protein
AER: Agro-ecological region
MRL: Maximum residual level
MAS: Marker-assisted selection
BSA: Bulk segregation analysis
NMR: Nuclear magnetic resonance
MMP: Matrix metalloproteinase
ORF: Open reading frame
NCBI: National Center for Biotechnology Information
PDB: Protein Data Bank
GRAVY: Grand average of hydropathicity
mRNA: Messenger ribonucleic acid
UTR: Untranslated region
EC: Epicatechin
EGCG: Epigallocatechingallate
