# Correction to: In silico structural homology modelling of EST073 motif coding protein of tea *Camellia sinensis* (L)

**DOI:** 10.1186/s43141-021-00291-3

**Published:** 2022-01-06

**Authors:** K. H. T. Karunarathna, N. H. K. S. Senathilake, K. M. Mewan, O. V. D. S. J. Weerasena, S. A. C. N. Perera

**Affiliations:** 1 Institute of Biochemistry, Molecular Biology and Biotechnology, University of Colombo, Colombo, Sri Lanka; 2 Current address: Department of Biosystems Technology, Faculty of Technology, University of Ruhuna, Matara, Sri Lanka; 3 Department of Biotechnology, Faculty of Agriculture and Plantation Management, Wayamba University of Sri Lanka, Makandura, Gonawila Sri Lanka; 4 Department of Agricultural Biology, Faculty of Agriculture, University of Peradeniya, Peradeniya, 20400 Sri Lanka

**Correction to: J Genet Eng Biotechnol 18, 32 (2020)**

**https://doi.org/10.1186/s43141-020-00038-6**

Following publication of the original article [1], the authors identified an error in the Acknowledgements section.

The updated Acknowledgements section is given below and the changes have been highlighted in **bold typeface**.
